# Supplementary material for: Blood metal levels and serum testosterone concentrations in male and female children and adolescents: NHANES 2011–2012
Source: PLoS One. 2019 Nov 7;14(11):e0224892. doi: 10.1371/journal.pone.0224892 (PMC6837506; doi:10.1371/journal.pone.0224892)
Supplement: Attachment — Submitted filename: PONE-D-19-17206_as reviewed. 08th July 2019.pdf [file pone.0224892.s005.pdf]

PLOS ONE

Blood metal levels and serum testosterone concentrations in male and female children and adolescents: NHANES 2011–2012

--Manuscript Draft--

**Manuscript Number:**	PONE-D-19-17206
**Article Type:**	Research Article
**Full Title:**	Blood metal levels and serum testosterone concentrations in male and female children and adolescents: NHANES 2011–2012
**Short Title:**	Blood metal levels and serum testosterone concentrations in male and female children and adolescents
**Corresponding Author:**	Rongkui Hu<br>Affiliated Hospital of Nanjing University of Chinese Medicine<br>Nanjing, CHINA
**Keywords:**	Metals; testosterone; National Health and nutrition examination survey (NHANES); children; adolescents
**Abstract:**	Environmental exposure to metals is ubiquitous while its relation to androgen hormone levels is not well understood, especially in children and adolescents. This study aimed to explore the relationship between blood metal concentrations and serum total testosterone (TT) levels in 6–19-year-old children and adolescent participants in the National Health and Nutrition Examination Survey (NHANES) 2011–2012. Multivariable linear regression models were employed to estimate the associations between log-transformed serum TT levels and quartiles or tertiles of blood lead, cadmium, total mercury, selenium, and manganese in male and female children (age 6-11years) and adolescents (age 12-19 years). We established that the blood cadmium and manganese levels were associated with significantly higher serum TT in the female adolescents. Additionally, the blood selenium levels in male adolescents were related to significantly higher serum TT; conversely, higher levers of blood selenium were associated with lower serum TT in the female children. No significant associations between blood lead or total mercury levels and TT were observed in children or adolescents of either sex. These findings suggest that metal exposure may positively or negatively affect the level of serum TT in the children or adolescents. Further research is required to confirm and extend our present findings.
**Order of Authors:**	Qi Yao
	Ge Zhou
	Meilin Xu
	Jianguo Dai
	Ziwei Qian
	Zijing Cai
	Luyao Zhang
	Yong Tan
	Rongkui Hu
**Additional Information:**	
**Question**	**Response**
**Financial Disclosure**<br><br>Enter a financial disclosure statement that describes the sources of funding for the work included in this submission. Review the submission guidelines for detailed	Yes

Powered by Editorial Manager® and ProduXion Manager® from Aries Systems Corporation

requirements. View published research articles from *PLOS ONE* for specific examples.<br><br>This statement is required for submission and **will appear in the published article** if the submission is accepted. Please make sure it is accurate.<br><br>**Unfunded studies**<br>Enter: *The author(s) received no specific funding for this work.*<br><br>**Funded studies**<br>Enter a statement with the following details:<br>• Initials of the authors who received each award<br>• Grant numbers awarded to each author<br>• The full name of each funder<br>• URL of each funder website<br>• Did the sponsors or funders play any role in the study design, data collection and analysis, decision to publish, or preparation of the manuscript?<br>• **NO** - Include this sentence at the end of your statement: *The funders had no role in study design, data collection and analysis, decision to publish, or preparation of the manuscript.*<br>• **YES** - Specify the role(s) played.<br><br>* typeset	
**Competing Interests**<br><br>Use the instructions below to enter a competing interest statement for this submission. On behalf of all authors, disclose any competing interests that could be perceived to bias this work—acknowledging all financial support and any other relevant financial or non-financial competing interests.<br><br>This statement **will appear in the published article** if the submission is accepted. Please make sure it is accurate. View published research articles from *PLOS ONE* for specific examples.	The authors have declared that no competing interests exist.

**NO authors have competing interests**<br><br>Enter: *The authors have declared that no competing interests exist.*<br><br>**Authors with competing interests**<br><br>Enter competing interest details beginning with this statement:<br><br>*I have read the journal's policy and the authors of this manuscript have the following competing interests: [insert competing interests here]*<br><br>* typeset	
**Ethics Statement**<br><br>Enter an ethics statement for this submission. This statement is required if the study involved:<br><br>• Human participants<br>• Human specimens or tissue<br>• Vertebrate animals or cephalopods<br>• Vertebrate embryos or tissues<br>• Field research<br><br>Write "N/A" if the submission does not require an ethics statement.<br><br>General guidance is provided below. Consult the submission guidelines for detailed instructions. **Make sure that all information entered here is included in the Methods section of the manuscript.**	We analyzed the data from NHANES 2011–2012.NHANES is a cross-sectional, U.S.-representative survey conducted annually by the Centers for Disease Control and Prevention (CDC).NHANES received approval from the NCHS Ethics Review Board, and informed consent was obtained for all participants.

Powered by Editorial Manager® and ProduXion Manager® from Aries Systems Corporation

**Format for specific study types**<br><br>**Human Subject Research (involving human participants and/or tissue)**<br>• Give the name of the institutional review board or ethics committee that approved the study<br>• Include the approval number and/or a statement indicating approval of this research<br>• Indicate the form of consent obtained (written/oral) or the reason that consent was not obtained (e.g. the data were analyzed anonymously)<br><br>**Animal Research (involving vertebrate animals, embryos or tissues)**<br>• Provide the name of the Institutional Animal Care and Use Committee (IACUC) or other relevant ethics board that reviewed the study protocol, and indicate whether they approved this research or granted a formal waiver of ethical approval<br>• Include an approval number if one was obtained<br>• If the study involved *non-human primates*, add *additional details* about animal welfare and steps taken to ameliorate suffering<br>• If anesthesia, euthanasia, or any kind of animal sacrifice is part of the study, include briefly which substances and/or methods were applied<br><br>**Field Research**<br><br>Include the following details if this study involves the collection of plant, animal, or other materials from a natural setting:<br>• Field permit number<br>• Name of the institution or relevant body that granted permission	
**Data Availability**<br><br>Authors are required to make all data underlying the findings described fully available, without restriction, and from the time of publication. PLOS allows rare exceptions to address legal and ethical concerns. See the PLOS Data Policy and FAQ for detailed information.	Yes - all data are fully available without restriction

A Data Availability Statement describing where the data can be found is required at submission. Your answers to this question constitute the Data Availability Statement and **will be published in the article**, if accepted.<br><br>**Important:** Stating 'data available on request from the author' is not sufficient. If your data are only available upon request, select 'No' for the first question and explain your exceptional situation in the text box.<br><br>Do the authors confirm that all data underlying the findings described in their manuscript are fully available without restriction?	
**Describe where the data may be found in full sentences. If you are copying our sample text, replace any instances of XXX with the appropriate details.**<br><br>• If the data are **held or will be held in a public repository**, include URLs, accession numbers or DOIs. If this information will only be available after acceptance, indicate this by ticking the box below. For example: *All XXX files are available from the XXX database (accession number(s) XXX, XXX.*).<br>• If the data are all contained **within the manuscript and/or Supporting Information files**, enter the following: *All relevant data are within the manuscript and its Supporting Information files.*<br>• If neither of these applies but you are able to provide **details of access elsewhere**, with or without limitations, please do so. For example:<br><br>*Data cannot be shared publicly because of [XXX]. Data are available from the XXX Institutional Data Access / Ethics Committee (contact via XXX) for researchers who meet the criteria for access to confidential data.*<br><br>*The data underlying the results presented in the study are available from (include the name of the third party*	All relevant data are within its Supporting Information files.

and contact information or URL).<br>• This text is appropriate if the data are owned by a third party and authors do not have permission to share the data.<br><br>* typeset	
Additional data availability information:	

ms Click here to download Manuscript ms.docx

Blood metal levels and serum testosterone concentrations in male and female children and adolescents: ~~NHANES 2011–2012~~

Qi Yao[1], Ge Zhou[2], Meilin Xu[3], Jianguo Dai[1], Ziwei Qian[1], Zijing Cai[1], Luyao Zhang[1], Yong Tan[2*], Rongkui Hu[2*]

Q.Y., G.Z. contributed equally to this work.

1: Department of Pathology and Pathophysiology, School of Medicine and Life Sciences, Nanjing University of Chinese Medicine, 138 Xianlin Road, Nanjing 210023, Jiangsu Province, China.

2: Department of Reproductive Medicine, Affiliated Hospital of Nanjing University of Chinese Medicine, Jiangsu Province Hospital of Chinese Medicine, 155 Hanzhong Road, Nanjing 210046, Jiangsu Province, China.

3：Medical department life science China, GE healthcare China, No.1 Tongji south road economic and technological development area, Beijing, 100176, China

Corresponding author: Profess Yong Tan, Department of Reproductive Medicine, Affiliated Hospital of Nanjing University of Chinese Medicine, Jiangsu Province Hospital of Chinese Medicine, 155 Hanzhong Road, Nanjing, 210046, Jiangsu Province, China. (E-mail: xijun1025@163.com)

Corresponding author: Associate Senior Doctor Rongkui Hu, Department of Reproductive Medicine, Affiliated Hospital of Nanjing University of Chinese Medicine, Jiangsu Province Hospital of Chinese Medicine, 155 Hanzhong Road, Nanjing, 210046, Jiangsu Province, China. (E-mail: xiangyu198110@163.com)

Keywords: Metals; testosterone; National Health and nutrition examination survey (NHANES); children; adolescents;

Abstract

Environmental exposure to metals is ubiquitous while its relation to androgen hormone levels is not well understood, especially in children and adolescents. This study aimed to explore the relationship between blood metal concentrations and serum total testosterone (TT) levels in 6–19-year-old children and adolescent participants in the National Health and Nutrition Examination Survey (NHANES) 2011–2012. Multivariable linear regression models were employed to estimate the associations between log-transformed serum TT levels and quartiles or tertiles of blood lead, cadmium, total mercury, selenium, and manganese in male and female children (age 6-11years) and adolescents (age 12-19 years). We established that the blood cadmium and manganese levels were associated with significantly higher serum TT in the female adolescents. Additionally, the blood selenium levels in male adolescents were related to significantly higher serum TT; conversely, higher levers of blood selenium were associated with lower serum TT in the female children. No significant associations between blood lead or total mercury levels and TT were observed in children or adolescents of either sex. These findings suggest that metal exposure may positively or negatively affect the level of serum TT in the children or adolescents. Further research is required to confirm and extend our present findings.

1. Introduction

Testosterone (T) is a principal sex hormone needed for the normal physiological processes at all life stages. In males, T is essential to the development and maintenance of secondary sexual traits [1-2]. T also influences bone mass, muscle strength, mood, and intellectual capacity [1-2]. In females, T is also of a crucial importance to bone density and is necessary for the normal ovarian and sexual function, libido, energy, and cardiovascular and cognitive functions [3-4].

The imbalance of serum T levels is leads to reproductive dysfunction at multiple life stages in both sexes. Low T levels are related to reduced semen quality in men [5, 6], increased genital malformations [7], and changes in the time of onset and/or progression of puberty [8-9]. On the other hand, high T levels are linked to polycystic ovary syndrome (PCOS) in females [10], increased gential system cancer [11, 12], and altered pubertal development [13, 14]. Therefore, research on the factors affecting the T levels in both sexes is meaningful.

The impact of environmental contaminants on T levels and the related reproductive health features has been an area of intense investigation in recent years. The general population is exposed every day to low concentrations of metals through the consumption of water, foods, supplements, or inhalation of air. Due to the widespread human exposure to metals, a growing concern exists about the adverse reproductive health effects. Toxic metal elements, such as cadmium [15-16], lead [17], and mercury [18] can cause reproductive toxicity in rats at relatively low levels. Essential trace elements, such as manganese [19] and selenium [20], have also been linked to

impaired T levels at levels above specific values in animals. The results of the epidemiologic studies exploring the associations between nonoccupational exposure to these metals and T levels are inconsistent. For example, Ali et al. reported a significantly positive association between blood cadmium and T levels in 438 postmenopausal Swedish women [21]. However, Chen et al. observed no association between blood cadmium and T levels in 1,589 postmenopausal Chinese women [22]. In addition, Interdonato et al. reported significantly negative association between urinary cadmium exposure and T levels in 111 male adolescents living in the Milazzo-Valle del Mela area [23], whereas the data of the Third National Health and Nutrition Examination Survey (NHANES III) indicated no significant associations between urinary cadmium and T levels in U.S. adult males [24]. The inconsistencies in the findings of these investigations may be due to differences in their study designs and subpopulation, as well as to small sample sizes or inadequate control of confounders such as the diurnal and seasonal variations in serum T. The diurnal and seasonal variations contribute to wide-range fluctuations in male serum T levels [25-26]. For example, the diurnal variations lead to a peak in the serum T levels in the early morning followed by a progressive decline to the nadir in the evening. Nadir values are approximately 15% lower than the peak morning values, but they may even vary by as much as 50% in younger males [27].

In this study, we investigated the potential associations between blood metal (cadmium, lead, mercury, manganese, and selenium) levels and that of circulating serum total T (TT) in a nationally representative general population sample of

6–11-year-old children and 12–19-year-old adolescents (in the United States).

2. Materials and Methods

2.1 Study population

We analyzed the data from NHANES 2011–2012. NHANES is a cross-sectional, U.S.-representative survey conducted annually by the Centers for Disease Control and Prevention (CDC). The goal of NHANES is to assess the health and nutritional status of the general U.S. population. The data were collected by questionnaire surveys, household interviews, physical examinations, and laboratory tests. We analyzed data from a subset of male children (age 6–11 years), male adolescents (age 12–19 years), female children (age 6–11 years), and female adolescents (age 12–19 years). Participants with missing data of their blood metal levels, serum testosterone, and covariates were excluded from the analysis. In the final sample size, we assessed the data from 431 male children, 493 male adolescents, 426 female children, and 470 female adolescents. NHANES received approval from the NCHS Ethics Review Board, and informed consent was obtained for all participants.

2.2 **Serum TT**

Serum TT levels were analyzed by isotope-dilution liquid chromatography–tandem mass spectrometry Information regarding the reliability, validation, and quality control of serum TT levels is presented in details in the NHANES laboratory methods (http://www.cdc.gov/nchs/data/nhanes/nhanes_11_12/TST_G_met.pdf). Serum TT was log-transformed for analyses, because the distribution of this variable was skewed

left.

2.3 Blood metals

Whole blood lead, cadmium, total mercury, manganese, and selenium concentrations were determined using inductively coupled plasma mass spectrometry based on the quadrupole ICP-MS technology. The methodological details of the detection and measurements of the blood metal levels are described in the NHANES laboratory methods. (http://www.cdc.gov/nchs/data/nhanes/nhanes_11_12/TST_G_met.pdf). The detection limit for all analytes was constant in the data set. The lower detection limit for lead was 0.25 μg/dL, whereas it was 0.16 μg/L for cadmium, 30 μg/L for selenium, 1.06 μg/L for manganese, and 0.16 μg/L for total mercury. Values below the LOD were imputed by LOD divided by the square root of 2, which provided an imputed value for the individuals who had exposure measurement levels below the LOD.

2.4 Covariates

We examined the following as potential confounding variables: age, race/ethnicity, poverty income ratio (PIR), obesity, season of collection, time of venipuncture, and serum cotinine as a biomarker of exposure to environmental tobacco smoke. Race/ethnicity was categorized as Non-Hispanic White, Non-Hispanic Black, Hispanic (Mexican American and other Hispanic black), and other. PIR represents the calculated ratio of the family income to the poverty threshold. Children and adolescents were classified as normal/underweight, overweight, or obese according to their age and sex, in compliance with the criteria defined by NHANES (Body

Measures File; http:// wwwn.cdc.gov/Nchs/Nhanes/2011-2012/BMX_G.htm). In our analyses, we combined underweight and normal weight in one category. The season of collection was obtained from the NHANES demographic data pertaining to the six-month period when the examination was conducted, which was then classified into two categories, including November 1st–April 30th and May 1st–October 31st. Time of venipuncture, which can be found in the NHANES Fasting Questionnaire File, was categorized as morning, afternoon, or evening sessions. Serum continine was log-transformed.

Statistical Analysis

All statistical analyses were performed using Empower (R) (www.empowerstats.com, X&Ysolutions, inc.Boston, MA) and R (http://www.R-project.org) software. Descriptive statistics of participant demographics and concentrations of the analytes was calculated. Metals with >75% of the samples>LOD were categorized into quartiles. The grouping cut-point was determined for cadmium by the percentage of samples LOD. The low group consisted of <LOD, whereas the medium and high groups consisted of equal-sized bins among the detected values. We employed multivariable linear regression to calculate the adjusted β-coefficients for the associations between categories for each metal and log-transformed serum TT among the participants. Because our dependent variable, serum TT, was log-transformed, the results were retransformed by exponentiation of the β - coefficients and presented as percent differences (equation: % change = [2^

beta-1]× 100).

In this study, we opted not to use sampling weights in our analysis because a weighted analysis with variables employed in the calculation of sampling weights included in the statistical models can lead to decreased precision of the effect estimates [28-29]. In NHANES 2011–2012, there was oversampling of certain racial, PIR, and age groups and, as a result, race, PIR, age in our statistical models, an unweighted analysis was more appropriate than a weighted analysis. This approach has been employed in other recent studies using data from NHANES [30-31]. *P*-values less than 0.05 were considered to be statistically significant.

3. Results

The characteristics of the study population by age and sex categories are presented in Table 1. As expected, serum TT concentrations were much higher in male adolescents than in female adolescents and in male adolescents than in male children. The median serum TT levels of male children, male adolescents, female children, and female adolescents were 3.16 ng/dL, 354.67 ng/dL, 4.86 ng/dL, and 23.95 ng/dL, respectively.

As can be seen in Table 2, ~~which shows~~ the distributions of blood metals by age and sex categories, blood manganese and selenium levels were >LOD for all samples. A large portion (98.6%–84.0%) of the samples had blood lead and total mercury levels >LOD, whereas 28.8%–58.7% of the samples had blood cadmium levels >LOD. The median concentrations of all blood metals were higher in adolescents than in

children, except for the levels of blood lead, and blood manganese among males, which were higher in children than in adolescents.

There were no statistically significant associations between serum TT and blood lead in any subgroup, and no evidence existed of consistent trends with increasing the quartiles of exposure (Table 3).

As can be observed in Table 4, serum TT was positively associated with blood cadmium in female adolescent participants, with a monotonic increase in the mean serum TT with increasing the tertiles of exposure (*p*-trend = 0.006, based on model 1) and significantly higher serum TT for the 2nd and 3rd tertiles (13.29%; 95% CI: 2.10%, 25.70%, and 16.47 %; 95% CI: 4.25%, 31.95%, respectively, based on model 1) compared with the lowest tertile. This pattern of association persisted following adjustment for age, BMI, race, serum cotinine, time of venipuncture, season of collection, ratio family income to poverty, blood lead, and blood selenium, and the *p*-trend was remained significant (*p*-trend = 0.002, based on model 2). Blood cadmium did not appear to be associated with serum TT in male and female children and male adolescent, and no evidence of consistent trends with increasing tertiles of exposure was established.

We found no statistically significant associations between serum TT and blood total mercury in any subgroup as well as no evidence of consistent trends with increasing quartiles of exposure (Table 5).

In male adolescent subjects, the mean serum TT level was higher for boys in the 3rd and 4th quartiles of blood selenium versus those in the 1st quartile, and the trend

p-value was significant (*p*-trend = 0.005, based on model 1) (Table 6). However, the quartile-specific increase was significant only for the 4th quartile (34.72%; 95% CI: 9.43%, 65.86%, based on model 1). The association were generally consistent with model1 after adjustment for age, BMI, race, serum cotinine, time of venipuncture, season of collection, ratio family income to poverty, blood cadmium, and blood lead, and the *p*-trend remained significant(*p*-trend =0.01, based on model 2). The mean serum TT was significantly lower for all quartiles of blood selenium than that of the lowest quartile in all population subgroups; the trend *p*-value was statistically significant for the female children (*p*-trend =0.03, based on model1). This pattern of association persisted following adjustment for age, BMI, race, serum cotinine, time of venipuncture, season of collection, ratio family income to poverty, blood cadmium, and blood lead, and the *p*-trend remained significant (*p*-trend = 0.02, based on model 2). Blood selenium did not appear to be associated with the serum TT level in male children and female adolescents, and no evidence of consistent trends with increasing quartiles of exposure was established.

As seen in Table 7, according to model 1, TT was significantly higher in all quartiles of blood manganese than in the lowest quartile in all population subgroups. The trend *p*-value was significant for female adolescents (*p*-trend = 0.02, based on model 1). After adjustment for age, the values of BMI, race, serum cotinine, time of venipuncture, season of collection, ratio of family income to poverty, and the *p*-trend remained significant (*p*-trend = 0.02, based on model 2). No significant associations were found between serum TT and blood manganese levels in male and female

children and male adolescents, and no evidence of consistent trends with increasing quartiles exposure was established.

4. Discussion

The aim of this study was to investigate the associations between blood metal (lead, cadmium, total mercury, manganese, and selenium) and serum TT levels in male and female children and adolescents. In the present cross-sectional analysis of data from NHANES 2011–2012, both blood cadmium and blood manganese levels were positively associated with serum TT levels in female adolescents. Additionally, blood selenium was positively associated with serum TT levels in male adolescents. In contrast, in female children, the high levers of blood selenium were associated with lower serum TT. No significant association between blood lead or total mercury and serum TT levels was found in any of the four population subgroups.

In the present investigation, we established no association between blood lead or cadmium and serum TT levels among children and adolescent males. In a previous study, Meeker et al. found no association between blood lead and serum T levels in 219 adult men recruited at two infertility clinics in Michigan, USA. The investigators found a positive association between blood cadmium and serum TT levels, which is in contrast to the results of the present study. In the analysis conducted by Meeker et al., the median blood metal levels were 1.5 μg/dL for lead and 2 μg/L for cadmium [32]. In another large examination using an NHANES (1999–2004) sample, Kresovich et al. established no association between blood cadmium and serum TT levels in 869 adult

men. The investigators found a positive association between blood lead and serum TT levels. Additionally, Kresovich et al. determined median blood metal levels of 2.0 µg/dL for lead and of 0.4 µg/L for cadmium [33]. The findings of the current study are comparable to those of previous analyses in terms of certain controlled characteristics, such as identical demographic age, race, and ratio of family income to poverty. Moreover, adjustments for BMI, diurnal and seasonal variations of serum T, and cotinine, selenium, and lead or cadmium exposure were also similarly conducted. Differences in cadmium or lead levels might have been the cause of the inconsistency between our results and those of previous research. We found that the blood lead and cadmium levels were much lower than those established in the investigations of Kresovichet al. and Meeker et al. The median metal levels determined in this study were 0.72µg/dL and 0.66µg/dL for blood lead and 0.11 µg/L and 0.16µg/L for blood cadmium in children and adolescent males, respectively.

To our best knowledge, this is the first report on the relationships between cadmium exposure and circulating T levels in adolescent female subjects. In this study, we observed a significant positive relationship between blood cadmium and circulating TT levels in 12–19-year-old girls. Cigarette smoking is a major source of cadmium exposure[34]. Kandel reported that T levels and smoking were positively correlated in mothers during their pregnancy and adult daughters [35]. Martin et al. reported that T levels were positively correlated with cigarette use in the last 30 days in young adult females [36]. In the aforementioned publication, three possible explanations were discussed for this increase in testosterone levels. First, previous

studies have shown that cadmium mimics estrogenic activities [37]. The enhancement of estrogenic activities caused by cadmium exposure might have influenced the mechanism that maintains the estrogen-androgen balance. Second, cadmium is also possible to have interfered with the LH-induced P450 aromatase activity responsible for the conversion of testosterone to estradiol. Das and Mukherjee showed that LH-stimulated P450 aromatase activity and P450arom gene expression in carp ovarian follicles were significantly inhibited by $CdCl_2$ [38]. Furthermore, Ali et al. observed a significant inverse association between blood cadmium and estradiol/testosterone ratio in 438 postmenopausal Swedish women [39]. Unfortunately, gonadotropins and estradiol were not measured in the 2011–2012 NHANES survey.

We observed a moderate but statistically significant positive association between blood manganese and serum TT levels in female adolescents. As established in several animal studies included in the review conducted by Dees, the exposure to lower, but still elevated levels manganese caused a release of hypothalamic luteinizing hormone-releasing hormone (LHRH). The effects of manganese on the LHRH-releasing system are consistent with the elevated serum levels of LH, FSH, and gonadal steroid in both sexes [40]. Therefore, this relationship might be an explanation of the mechanism by which exposure manganese is involved in the estrogen–androgen balance in female adolescents. However, the findings of this study showed no association between blood manganese and serum TT levels in male adolescents. The reason for these gender differences might have been caused by the

diverse manganese metabolism of the two sexes since male rats clearing the element over twice faster than females [41-42]. Furthermore, in this study, the median blood manganese levels of adolescent males (9.33μg/L) were lower than that of adolescent females (10.84μg/L).

We also observed a moderate but statistically significant positive association between blood selenium and serum TT levels in male adolescents. Selenium is an essential trace element especially required for maintenance of spermatogenesis and male fertility [43]. In previous studies, appropriate selenium levels appeared to exert a positive influence on the Leydig cells, thus influencing the secretion of testosterone [44]. Epidemiologic study also revealed that supplemental selenium in infertile men serum T tended to increase[45].

Certain limitations of the present study should be acknowledged. First, NHANES is a survey with a cross-sectional design, which restricts the proper interpretation of the causal associations between the metal exposure and serum TT. Second, we did not have data on free T, SHBG, and other hormones or markers that might have provided clues for the mechanisms and/or sites of action of blood metals. Third, the group of 12-to-19-year-old boys and girls classified as adolescents may have included a mixture of children who were pre- and postpubescent. Lastly, other confounding factors might also have been available that we did not evaluate in our analysis, including exposure to other metals such as iron.

5. Conclusions

The results from this study suggest that the blood levels of certain metals were

associated with altered serum TT concentrations in male and female children and adolescents, included in NHANES 2011–2012. Our findings pertain to low-level environmental metals exposure and may not be generalizable to environmental or occupational settings involving higher dosages of metals. Additionally, the altered T levels were found to be linked to a wide range of adverse health effects, but additional human epidemiology studies, as well as mechanistic studies, are needed to confirm the results of our analysis.

Author Contributions： Q.Y.: Research idea and participation in related article writing. R. Hu.: Project administration. G.Z., M.X., J.D., Z.Q., Z.C., L.Z. and Y. T.: Interpreted the results. All authors read and approved the final manuscript.

Funding: Work supported by grants from the National Natural Science Founds for Young Scholar (Grant No. 81704164), Natural Science Foundation of Jiangsu Province (Grant No. BK20151043), Six Talent Peaks Project in Jiangsu Province (Grant No. WSN-044) and 333 Talent Project in Jiangsu Province (Grant No. 2016Ⅲ-3288).

Achnowledgments: We are grateful for the editors and reviewers.

Conflicts of Interest: The authors declare no conflict of interest.

References

1. Surampudi PN, Wang C, Swerdloff R. Hypogonadism in the aging male diagnosis, potential benefits, and risks of testosterone replacement therapy. Int J Endocrinol. 2012; 2012: 625434.

2. Bassil N, Alkaade S, Morley JE. The benefits and risks of testosterone replacement therapy: a review. Ther Clin Risk Manag. 2009; 5(3): 427-448.

3. Pluchino N, Carmignani A, Cubeddu A, Santoro A, Cela V, Errasti T. Androgen therapy in women: for whom and when. Arch Gynecol Obstet. 2013; 288(4): 731-737.

4. Davis SR, Wahlin-Jacobsen S. Testosterone in women--e clinical significance. Lancet Diabetes Endocrinol. 2015; 3(12): 980-992.

5.Carlsen E, Giwercman A, Keiding N, Sakkkebaek NE. Evidence for decreasing quality of semen during past 50 years.BMJ. 1992; 305(6854): 609-613.

6. Rolland M1, Le Moal J, Wagner V, Royère D, thDe Mouzon J. Decline in semen concentration and morphology in a sample of 26,609 men close to general population between 1989 and 2005 in France. Hum Reprod. 2013; 28(2): 462-470.

7. Main KM, Skakkebaek NE, Virtanen HE, Toppari J. Genital anomalies in boys and the environment. Best Pract Res Clin Endocrinol Metab. 2010; 24(2): 279-289.

8. Euling SY, Herman-Giddens ME, Lee PA, Selevan SG, Juul A, Sørensen TI, Dunkel L, Himes JH, Teilmann G, Swan SH. Examination of US puberty-timing data from 1940 to 1994 for secular trends: panel findings. Pediatrics. 2008; 121 (Suppl 3): S172-191.

9. Zawatski W, Lee MM. Male pubertal development: are endocrine-disrupting compounds shifting the norms? J Endocrinol. 2013; 182(2):R1-R12.
10. Escobar-Morreale HF. Polycystic ovary syndrome: definition, aetiology, diagnosis and treatment. Nat Rev Endocrinol 2018; 14(5): 270-284.
11. Klap J, Schmid M, Loughlin KR. The relationship between total testosterone levels and prostate cancer: a review of the continuing controversy. J Urol. 2015; 193(2): 403-413.
12. Gann PH, Hennekens CH, Ma J, Longcope C, Stampfer MJ. Prospective study of sex hormone levels and risk of prostate cancer.J Natl Cancer Inst. 1996; 88(16): 1118-1126.
13. Cole TJ, Ahmed ML, Preece MA, Hindmarsh P, Dunger DB. The relationship between Insulin-like Growth Factor 1, sex steroids and timing of the pubertal growth spurt. Clin Endocrinol (Oxf). 2015; 82(6): 862-869.
14. Cabrera SM, Rogol AD. Testosterone exposure in childhood: discerning pathology from physiology. Expert Opin Drug Saf. 2013; 12(3): 375-388.
15. Lacorte LM, Rinaldi JC, Justulin LA Jr, Delella FK, Moroz A, Felisbino SL. Cadmium exposure inhibits MMP2 and MMP9 activities in the prostate and testis. Biochem Biophys Res Commun. 2015; 457(4):538-541.
16. Belani M, Purohit N, Pillai P, Gupta S, Gupta S. Modulation of steroidogenic pathway in rat granulosa cells with subclinical Cd exposure and insulin resistance: an impact on female fertility. Biomed Res Int. 2014; 2014: 460251.
17. Ronis MJ, Badger TM, Shema SJ, Roberson PK, Shaikh F. Reproductive toxicity

and growth effects in rats exposed to lead at different periods during development. Toxicol Appl Pharmacol. 1996; 136(2): 361-371.

18.Rizzetti DA, Martinez CS, Escobar AG, da Silva TM, Uranga-Ocio JA, Peçanha FM, Vassallo DV, Castro MM, Wiggers GA. Egg white-derived peptides prevent male reproductive dysfunction induced by mercury in rats. Food Chem Toxicol. 2017; 100: 253-264.

19. Mohammed AT, Ebraheim LLM, Metwally MMM. Ebselen can Protect Male Reproductive Organs and Male Fertility from Manganese Toxicity: Structural and Bioanalytical Approach in a Rat Model. Biomed Pharmacother. 2018; 102:739-748.

20. Liu L, He Y, Xiao Z, Tao W1 Zhu J, Wang B, Liu Z, Wang M. Effects of Selenium Nanoparticles on Reproductive Performance of Male Sprague-Dawley Rats at Supranutritional and Nonlethal Levels. Biol Trace Elem Res. 2017; 180(1): 81-89.

21. Ali I, Engström A, Vahter M, Skerfving S, Lundh T, Lidfeldt J, Samsioe G, Halldin K, Åkesson A. Associations between cadmium exposure and circulating levels of sex hormones in postmenopausal women. Environ Res. 2014; 134:265-269.

22. Chen C, Wang N, Nie X, Han B, Li Q, Chen Y, Zhai H, Zhu C, Chen Y, Xia F, Lu M, Lin D, Lu Y. Blood Cadmium Level Associates with Lower Testosterone and Sex Hormone-Binding Globulin in Chinese men: from SPECT-China Study, 2014. Biol Trace Elem Res. 2016;171(1): 71-78.

23. Interdonato M, Pizzino G, Bitto A, Galfo F, Irrera N, Mecchio A, Pallio G, Ramistella V, De Luca F, Santamaria A, Minutoli L, Marini H, Squadrito F, Altavilla D. Cadmium delays puberty onset and testis growth in adolescents. Clin Endocrinol

(Oxf). 2015; 83(3):357-362.

24. Menke A, Guallar E, Shiels MS, Rohrmann S, Basaria S, Rifai N, Nelson WG, Platz EA. The association of urinary cadmium with sex steroid hormone concentrations in a general population sample of US adult men.BMC Public Health. 2008; 8:72.

25. Brambilla DJ, Matsumoto AM, Araujo AB, McKinlay JB.The effect of diurnal variation on clinical measurement of serum testosterone and other sex hormone levels in men. J Clin Endocrinol Metab. 2009; 94(3): 907-913.

26. Cunningham GR, Toma SM. Clinical review: Why is androgen replacement in males controversial? J Clin Endocrinol Metab. 2011; 96(1):38-52.

27. Paduch DA, Brannigan RE, Fuchs EF, Kim ED, Marmar JL, Sandlow JI. The laboratory diagnosis of testosterone deficiency. Urology. 2014; 83(5): 980-988.

28. Korn EL, Graubard BI. Epidemiologic studies utilizing surveys: accounting for the sampling design. Am J Public Health 1991; 81(9): 1166-1173.

29. Silver MK, Lozoff B, Meeker JD. Blood cadmium is elevated in iron deficient U.S. children: a cross-sectional study. Environ Health. 2013; 30(12): 117.

30. Lewis RC, Johns LE, Meeker JD. Serum Biomarkers of Exposure to Perfluoroalkyl Substances in Relation to Serum Testosterone and Measures of Thyroid Function among Adults and Adolescents from NHANES 2011-2012.Int J Environ Res Public Health. 2015; 12(6): 6098-6114.

31. Lewis RC, Meeker JD. Biomarkers of exposure to molybdenum and other metals in relation to testosterone among men from the United States National Health and

Nutrition Examination Survey 2011-2012. Fertil Steril. 2015; 103(1): 172-178.

32. Meeker JD, Rossano MG, Protas B, Padmanahban V, Diamond MP, Puscheck E, Daly D, Paneth N, Wirth JJ. Environmental exposure to metals and male reproductive hormones: circulating testosterone is inversely associated with blood molybdenum. Fertil Steril. 2010; 93(1): 130-140.

33. Kresovich JK, Argos M, Turyk ME. Associations of lead and cadmium with sex hormones in adult males. Environ Res. 2015; 142:25-33.

34. Agency for Toxic Substances and Disease Registry (ATSDR). Toxicological Profile for Cadmium. Atlanta, GA: U.S. Department of Health and Human Services, Public Health Service, 1999.

35. Kandel DB, Udry JR. Prenatal effects of maternal smoking on daughters' smoking: nicotine or testosterone exposure? Am J Public Health. 1999; 89(9):1377-1383.

36. Martin CA, Logan TK, Portis C, Leukefeld CG, Lynam D, Staton M, Brogli B, Flory K, Clayton RR. The association of testosterone with nicotine use in young adult females. Addict Behav. 2001; 26(2):279-283.

37. larke L, Singh B, Chepko G, Clarke R, Sholler PF, Lirio AA, Foss C, Reiter R, Trock B, Paik S, Martin MB. Cadmium mimics the in vivo effects of estrogen in the uterus and mammary gland. Nat Med. 2003; 9(8): 1081-1084.

38. Das S, Mukherjee D. Effect of cadmium chloride on secretion of 17β-estradiol by the ovarian follicles of common carp, Cyprinus carpio. Gen Comp Endocrinol. 2013; 181: 107-114.

39. Ali I, Engström A, Vahter M, Skerfving S, Lundh T, Lidfeldt J, Samsioe G,

Halldin K, Åkesson A. Associations between cadmium exposure and circulating levels of sex hormones in postmenopausal women. Environ Res. 2014; 134: 265-269.

40. Dees WL, Hiney JK, Srivastava VK. Influences of manganese on pubertal development. J Endocrinol. 2017; 235(1): R33-R42.

41. Zheng W, Kim H, Zhao Q. Comparative toxicokinetics of manganese chloride and methylcyclopentadienyl manganese tricarbonyl (MMT) in Sprague-Dawley rats. Toxicol Sci. 2000; 54(2): 295-301.

42. Oulhote Y, Mergler D, Bouchard MF. Sex- and age-differences in blood manganese levels in the U.S. general population: national health and nutrition examination survey 2011-2012.Environ Health. 2014:13: 87.

43. Ursini F, Heim S, Kiess M, Maiorino M, Roveri A, Wissing J, Flohé L. Dual function of the selenoprotein PHGPx during sperm maturation. Science. 1999; 285(5432): 1393-1396.

44. Shi L, Song R, Yao X, Ren Y. Effects of selenium on the proliferation, apoptosis and testosterone production of sheep Leydig cells in vitro. Theriogenology. 2017; 93:24-32.

45. Safarinejad MR, Safarinejad S. Efficacy of selenium and/or N-acetyl-cysteine for improving semen parameters in infertile men: a double-blind, placebo controlled, randomized study. J Urol. 2009; 181(2): 741-751.

Table 1. Characteristics of the 6–19-year-old children and adolescent participants in NHANES 2011–2012.

Parameter	Male children	Male adolescents	Female children	Female adolescents
n	431	493	426	470
Age (years)	9 (7-10)	15(14-17)	9 (7-10)	15(13-17)
Serum total testosterone (ng/dL)	3.16 (1.80-5.68)	354.67（208.08-496.58）	4.86 (2.69-10.29)	23.95（16.72-31.55）
Serum cotinine (ng/mL)	0.035 (0.011-0.242)	0.038（0.011-0.431）	0.039 (0.011-0.181)	0.028（0.011-0.210）
Ratio family income to poverty	1.26(0.74-2.70)	1.57（0.86-3.40）	1.30 (0.70-2.78)	1.40（0.69-2.96）
Obesity[a]				
Normal/underweight	263 (61.02%)	309 (62.68%)	266 (62.44%)	297（63.19%）
Overweight	66 (15.31%)	77 (15.62%)	68 (15.96%)	76（16.17%）
Obese	102 (23.67%)	107 (21.70%)	92 (21.60%)	97（20.64%）
Race/ethnicity				
Non-Hispanic White	113 (26.22%)	119 (24.14%)	98(23.01%)	105（22.34%）
Non-Hispanic black	116 (26.91%)	154 (31.24%)	128(30.05%)	141（30.00%）
Hispanic	145 (33.64%)	138 (27.99%)	129(30.28%)	144（30.64%）
Other	57 (13.23%)	82 (16.63%)	71(16.67%)	80（17.02%）
Session time of venipuncture				
Morning	177 (41.07%)	243（49.29%）	207 (48.59%)	237（50.43%）
Afternoon	157 (36.43%)	170（34.48%）	149 (34.98%)	156（33.19%）
Evening	97 (22.51%)	80（16.23%）	70 (16.43%)	77（16.38%）
Six-month period when the examination was performed				
1 November through 30 April	216 (50.12%)	237（48.29%）	221 (51.88%)	216 (45.96%)
1 May through 31 October	215 (49.88%)	256（51.93%）	205 (48.12%)	254 (54.04%）

Data are summarized as median (interquartile range) for continuous variables or as number with proportion for categorical variables.

[a]Children and adolescents were classified as normal/underweight, overweight, or obese according to their age and sex, as defined by NHANES (http:/wwwn.cdc.gov/Nchs/Nhanes/2011-2012/BMX _G.htm)

Table 2. ~~Distribution of~~ metal concentrations in the blood of the 6–19-year-old children and adolescent participan[illegible] in NHANES 2011–2012.

Parameter	Male children				Male adolescents			
n	431				493			
	N(%)<LOD	Geometric	median	interquartile	N(%)<LOD	Geometric	median	interquartile
Blood lead (µg/dL)	7 (1.6%)	0.76	0.72	0.52-1.02	11 (2.2%)	0.68	0.66	0.47-0.96
Blood cadmium (µg/L)	311 (72.2%)	0.13	0.11	0.11-0.16	237 (48.1%)	0.17	0.16	0.11-0.23
Blood mercury, total (µg/L)	69 (16.0%)	0.35	0.34	0.20-0.59	48 (9.7%)	0.47	0.43	0.23-0.79
Blood selenium (µg/L)	0 (0%)	174	175	163-188	0 (0%)	187	187	174-202
Blood manganese (µg/L)	0 (0%)	9.79	9.72	7.86-11.84	0 (0%)	9.43	9.33	7.62-11.54
**Parameter**	**Female children**				**Female adolescents**			
n	426				470			
	N(%)<LOD	Geometric	median	interquartile	N(%)<LOD	Geometric	median	interquartile
Blood lead (µg/L)	6 (1.4%)	0.68	0.65	0.48-0.93	27 (5.8%)	0.47	0.47	0.35-0.63
Blood cadmium (µg/L)	277 (65.0%)	0.14	0.11	0.11-0.18	194 (41.3%)	0.19	0.18	0.11-0.27
Blood mercury, total (µg/L)	61 (14.3%)	0.39	0.36	0.22-0.66	40 (8.5%)	0.50	0.49	0.26-0.83
Blood selenium (µg/L)	0 (0%)	177	177	165-189	0 (100%)	184	183	169-200
Blood manganese (µg/L)	0 (0%)	10.55	10.52	8.72-12.90	0 (100%)	10.75	10.84	8.64-13.26

Table 3. Percent differences (95% CI) in the serum TT by quartiles of blood lead exposure, NHANES, 2011–2012.

Blood lead (μg/dL)	Model 1[a]	Model 2[b]
Male children		
≤0.52	Reference	Reference
0.52-0.72	-2.10 (-31.95, 27.45)	4.25 (-24.83, 35.66)
0.72-1.02	-17.28 (-53.69, 10.96)	-13.29 (-48.45, 15.67)
＞1.02	-18.92 (-55.83, 10.19)	-18.10(-55.83, 11.73)
p-trend	0.12	0.13
Male adolescents		
≤0.47	Reference	Reference
0.47-0.66	-0.70 (-23.11, 21.42)	4.25 (-27.46,15.67)
0.66-0.96	18.10 (-3.53, 45.40)	10.96 (-9.43, 35.66)
＞0.96	5.70 (-14.87, 29.24）	-0.70 (-23.97, 21.42)
p-trend	0.29	0.72
Female children		
≤0.48	Reference	Reference
0.48-0.65	9.43 (-10.96, 32.87)	13.29 (-7.8, 37.55)
0.65-0.93	10.19 (-10.19, 34.72）	11.73 (-8.67, 36.60）
＞0.93	-0.70 (-23.11, 21.42)	0.35 (-22.26, 23.11)
p-trend	0.95	0.99
Female adolescents		
≤0.35	Reference	Reference
0.35-0.47	-12.51 (-26.58, 0.70)	-10.19 (-24.83, 2.81)
0.47-0.63	-4.97 (-18.92,7.92)	-1.40 (-15.67,11.73)
＞0.63	4.25 (-8.67,18.10)	4.97 (-8.67,19.74)
p-trend	0.35	0.32

[a] Adjusted for age (continuous), BMI (normal/underweight, overweight, and obese) and race-ethnicity (Non-Hispanic White, Non-Hispanic black, Hispanic, and other).

[b] Adjusted for variables in model 1 plus serum cotinine (log-transformed, continuous), time of venipuncture (morning, afternoon, and evening), season of collection (1 November through 30 April, 1 May through 31 October), ratio family income to poverty (continuous), blood cadmium (log-transformed, continuous), and blood selenium (log-transformed, continuous)

Table 4. Percent differences (95% CI) in serum TT by tertiles of blood cadmium exposure, NHANES, 2011–2012.

Blood cadmium (μg/L)	Model 1[a]	Model2[b]
Male children		
≤0.11	Reference	Reference
0.11-0.20	3.53(-25.70, 34.72)	10.19(-18.10,43.40)
＞0.20	-16.47 (-54.76,14.87)	-11.73 (-48.45,18.92)
p-trend	0.41	0.66
Male adolescents		
≤0.11	Reference	Reference
0.11-0.23	4.97 (-13.29, 23.97)	2.81 (-14.87, 21.42)
＞0.23	16.47 (-2.81,40.44)	11.73 (-8.67, 35.66)
p-trend	0.10	0.26
Female children		
≤0.11	Reference	Reference
0.11-0.20	0.70 (-18.10,20.58)	-1.40 (-20.58,18.10)
＞0.20	-2.81 (-24.83,18.10)	-2.81 (-24.83,18.10)
p-trend	0.84	0.78
Female adolescent		
≤0.11	Reference	Reference
0.11-0.25	13.29(2.10, 25.70)	14.08(2.81, 26.58)
＞0.25	16.47 (4.25,31.95)	19.25 (5.70, 36.60)
p-trend	0.006	0.002

[a] Adjusted for age (continuous), BMI (normal/underweight, overweight, and obese) and race-ethnicity (Non-Hispanic White, Non-Hispanic black, Hispanic and other), serum cotinine (log-transformed, continuous).
[b] Adjusted for variables in model 1 plus time of venipuncture (morning, afternoon, and evening), season of collection (1 November through 30 April, 1 May through 31 October), ratio family income to poverty (continuous), blood lead (log-transformed, continuous), and blood selenium (log-transformed, continuous)

Table 5. Percent differences (95% CI) in serum TT by quartiles of blood mercury exposure, NHANES, 2011–2012.

Blood mercury (μg/L)	Model 1[a]	Model2[b]
Male children		
≤0.20	Reference	Reference
0.20-0.34	14.87 (-13.29, 49.48)	10.19 (17.28, 42.41)
0.34-0.59	7.18 (-21.42, 38.51)	1.40 (-27.46, 31.4)
＞0.59	-4.97(-37.55, 25.70)	-12.51 (-43.73,17.28)
p-trend	0.68	0.36
Male adolescents		
≤0.23	Reference	Reference
0.23-0.43	18.92 (-2.1, 45.40)	19.75 (-8.67, 45.40)
0.43-0.79	6.44 (-14.87, 31.04)	8.06 (-12.51, 32.87)
＞0.79	13.29 (-8.67, 40.44)	10.96 (-10.19, 36.60)
p-trend	0.40	0.44
Female children		
≤0.22	Reference	Reference
0.22-0.36	7.92 (-12.51, 30.13)	5.70 (-14.08, 28.34)
0.36-0.66	-0.01 (-20.58, 20.58)	-2.81 (-23.97, 18.10)
＞0.66	23.11 (-1.40,49.48)	21.42 (-0.28, 47.43)
p-trend	0.09	0.13
Female adolescent		
≤0.26	Reference	Reference
0.26-0.49	-0.21 (-15.67, 10.19)	-0.70 (-14.08,12.51)
0.49-0.83	-0.70 (-14.08,12.51)	0.70 (-12.51,14.87)
＞0.83	-2.81 (-17.28,10.96)	1.4 0(-15.67,13.29)
p-trend	0.68	0.95

[a] Adjusted for age (continuous), BMI (normal/underweight, overweight, and obese) and race-ethnicity (Non-Hispanic White, Non-Hispanic black, Hispanic, and other).
[b] Adjusted for variables in model 1 plus serum cotinine (log-transformed, continuous), time of venipuncture (morning, afternoon, and evening), season of collection(1 November through 30 April, 1 May through 31 October), ratio family income to poverty (continuous)

Table 6. Percent differences (95% CI) in serum TT by quartiles of blood selenium exposure, NHANES, 2011–2012.

Blood selenium (μg/L)	Model 1[a]	Model 2[b]
Male children		
≤163	Reference	Reference
163-175	6.44 (-23.11, 39.47)	5.70(23.97, 37.55)
175-188	-7.92 (-39.47, 19.75)	-8.67 (-39.47,18.92)
＞188	4.25 (-25.70, 35.66)	6.44 (-22.26, 38.51)
p-trend	0.95	0.93
Male adolescents		
≤174	Reference	Reference
174-187	-1.40 (-23.97, 21.42)	-0.70 (-22.26, 21.42)
187-202	3.53(-18.10, 26.58)	1.40 (-19.75, 23.97)
＞202	34.72 (9.43, 65.86)	31.04 (7.18, 61.33)
p-trend	0.005	0.01
Female children		
≤165	Reference	Reference
165-177	-23.97 (-50.52,-2.82)	- 25.70(-51.57, -3.53)
177-189	- 22.26(-47.43,-0.70)	-24.83 (-50.52, -3.53)
＞189	- 24.83(-50.52,-2.81)	- 26.58(-53.69, -4.97)
p-trend	0.03	0.02
Female adolescents		
≤169	Reference	Reference
169-183	1.40(-11.73, 14.87)	4.25(-8.67, 18.10)
183-200	-8.67 (-23.11,4.25)	-7.18 (-21.42, 4.92)
＞200	- 6.44(-20.58, 6.44)	-4.25 (-18.10, 8.67）
p-trend	0.17	0.23

[a] Adjusted for age (continuous), BMI (normal/underweight, overweight, and obese) and race-ethnicity (Non-Hispanic White, Non-Hispanic black, Hispanic, and other).

[b] Adjusted for variables in model 1 plus serum cotinine (log-transformed, continuous), time of venipuncture (morning, afternoon, and evening), season of collection (1 November through 30 April, 1 May through 31 October), ratio family income to poverty (continuous), blood lead (log-transformed, continuous), and blood cadmium (log-transformed, continuous)

Table 7. Percent differences (95% CI) in serum TT by quartiles of blood manganese exposure, NHANES, 2011–2012.

Blood Manganese (μg/L)	Model 1 [a]	Model2 [b]
Male children		
≤7.86	Reference	Reference
7.86-9.72	-10.96 (-44.39,17.28)	-11.73(-44.39,15.67)
9.72-11.84	16.47 (-12.51, 52.63)	16.47 (-11.73,51.57)
＞11.84	-4.97(-38.51, 26.58)	-6.44 (-41.42,23.97)
p-trend	0.81	0.88
Male adolescents		
≤7.62	Reference	Reference
7.62-9.33	23.97(1.40, 51.57)	23.97 (2.10, 50.52)
9.33-11.54	15.67(-6.44, 42.41)	9.43 (-11.73, 34.72)
＞11.54	18.92 (-4.25,47.43)	16.47 (-5.70, 44.39)
p-trend	0.19	0.32
Female children		
≤8.72	Reference	Reference
8.72-10.52	-28.34 (-55.83, -5.70)	-24.83(-51.57, 2.81)
10.52-12.90	- 2.10(-24.83,18.92)	- 2.10(-23.97, 19.75)
＞12.90	-2.10(-26.58, 21.42)	-0.70(-24.83, 22.26)
p-trend	0.57	0.55
Female adolescents		
≤8.64	Reference	Reference
8.64-10.84	17.28 (3.53, 33.79)	18.10 (3.53, 33.79)
10.84-13.26	23.11 (8.67, 40.44)	23.11 (7.92, 40.44)
＞13.26	16.47 (2.10, 32.87)	16.47 (2.10, 32.87)
p-trend	0.02	0.02

[a] Adjusted for age (continuous) , BMI (normal/underweight, overweight, and obese) and race-ethnicity (Non-Hispanic White, Non-Hispanic black, Hispanic, and other).

[b] Adjusted for variables in model 1 plus serum cotinine (log-transformed, continuous), time of venipuncture (morning, afternoon, and evening), season of collection (1 November through 30 April, 1 May through 31 October), ratio family income to poverty (continuous).

Data

Click here to access/download
Supporting Information
Data.xls

Data name

Click here to access/download
Supporting Information
DATAname.xls
