# Supplementary material for: Blood metal levels and serum testosterone concentrations in male and female children and adolescents: NHANES 2011–2012
Source: PLoS One. 2019 Nov 7;14(11):e0224892. doi: 10.1371/journal.pone.0224892 (PMC6837506; doi:10.1371/journal.pone.0224892)
Supplement: Attachment — Submitted filename: Eman Reviewer.docx [file pone.0224892.s003.docx]

**Reviewer:**

**Recommendation:** Minor Revision

The current study showed that environmental exposure to metals may positively or negatively affect the level of serum TT in the children or adolescents. This study also points out that certain metals lead to imbalance of serum T levels in male and female children and adolescents, included in NHANES 2011–2012. Further experimental and clinical research is required in order to elucidate the substantial mechanisms by which changes in testosterone levels have undesired effects on human health . This manuscript eligible to be published in Journal PLOS ONE . However, some changes need to be performed before considering publications as follows:

**Comments:**

1. General comments: Define all abbreviations the first time they are used.  Abbreviations defined in the abstract should be redefined in the body of the manuscript.
2. The abbreviation for serum TT levels is not consistent throughout the manuscript,
3. There are many details are needed, For an example on the introduction and discussion include reference to many studies for which the model studied (human, rats, mice) is not identified and the route of exposure is not provided.
4. In the “Introduction and Discussion” section There are repetition in many paragraphs. Please avoid redundancies
5. In the “Discussion” section the authors need to explain the significance of their findings and the novelty of their study and should substantiate their findings with already available relevant scientific information.
6. Discussion is too long , hard to follow and could be better organized.
7. In the “Discussion” section 1 st paragraph ( line7). Please correct levers to levels
8. Parts of the Discussion section are particularly hard to understand. Professional assistance is recommended.
9. In result caption section . **Table 2.** please add table legand blew the table
