# Supplementary material for: Blood metal levels and serum testosterone concentrations in male and female children and adolescents: NHANES 2011–2012
Source: PLoS One. 2019 Nov 7;14(11):e0224892. doi: 10.1371/journal.pone.0224892 (PMC6837506; doi:10.1371/journal.pone.0224892)
Supplement: Attachment — Submitted filename: Review Report.docx [file pone.0224892.s004.docx]

**Review Report**

Manuscript number: PONE-D-19-17206

Title:

Blood metal levels and serum testosterone concentrations in male and female children and adolescents: NHANES 2011–2012

Dated: 09^th^ July 2019

The manuscript describes the data taken on metals levels in blood and their association with testosterone levels in children and adolescents. The authors have successfully correlated two metals concentrations (Cd and Mn) with testosterone concentrations in the subjects, highlighting the fact that environmental exposure to heavy metals may lead to altered testosterone levels in growing children. Overall the manuscript describes interesting findings, which are important to publish.

However, I have few questions in mind while reading this manuscript for review purpose, which the authors need to answer before the manuscript is accepted for publication, as detailed below:

**Title Revision:**

The title of the manuscript may be revised a little bit as, “A correlation between blood metal levels and serum testosterone concentrations in children and adolescents: NHANES 2011–2012”

**Data:**

The data were collected during 2011-2012, why publishing so late? Why not published earlier?

**Introduction**:

Gives adequate background of the problem under investigation

**Materials and methods:**

Study population is ok, sample size are also enough, but data seems to be quite old when collected during 2011-2012. I wonder why it was not published earlier. The authors need to provide justification for using so old data.

How metals to be studied in blood of patients were selected? Why not chromium was also studied?

**Results:**

The results have been presented in tabulated form, although analyzed statistically. However, it would be better if results could be expressed in the form of a couple of figures, for the reader to understand more clearly the association between metals levels and TT concentrations.

**Discussion**:

The results have been discussed in a proper way, also the association of a couple of metals have been developed and discussed. However, what is lacking in this section is where the subjects (patients) are getting these metals from? I mean where are these exposed to heavy metals? What are the sources of these metals and also the route (inhalation, feeing or what?). The authors need to include these points in the discussion section.

**Recommendation**

The manuscript can be published but needs revision as suggested in the detailed review report attached. I have also made few text changes in the manuscript PDF file, which needs to be conveyed to the authors, for revision also.

************************************************************************
