# Supplementary material for: Blood metal levels and serum testosterone concentrations in male and female children and adolescents: NHANES 2011–2012
Source: PLoS One. 2019 Nov 7;14(11):e0224892. doi: 10.1371/journal.pone.0224892 (PMC6837506; doi:10.1371/journal.pone.0224892)
Supplement: Attachment — Submitted filename: Response to Reviewers.docx [file pone.0224892.s007.docx]

Dear editor:

Thank you very much for your letter and further instructions. We have carefully studied the comments and made corrections which we hope would meet with approval. Revisions are marked in red in the paper. The main corrections in the paper and responses to the reviewer’s comments are listed below.

Sincerely yours,

Rongkui Hu, M.M.

Department of Reproductive Medicine, Affiliated Hospital of Nanjing University of Chinese Medicine, Jiangsu Province Hospital of Chinese Medicine, 155 Hanzhong Road, Nanjing, 210046, Jiangsu Province, China.

E-mail: xiangyu198110@163.com

**Responses to the reviewers**

We sincerely thank all the reviewers for their positive and constructive comments and suggestions.

**Reviewer #4:**

**1. Response to comment:** Page 6, section 2.5. Add” The selection of these confounders was based on literatures” in front of “ Race/ethnicity was… ”

Response: We have accepted your good suggestion, Please see Page 6，section 2.5.
